# The expression of nicotinic receptor alpha7 during cochlear development

**DOI:** 10.1002/brb3.84

**Published:** 2012-08-23

**Authors:** Scott W Rogers, Elizabeth J Myers, Lorise C Gahring

**Affiliations:** 1 Salt Lake City VA Geriatric Research, Education and Clinical Center, University of UtahSalt Lake City, Utah, 84132; 2 Department of Neurobiology and Anatomy, University of Utah School of MedicineSalt Lake City, Utah, 84132; 3 Division of Geriatrics, Department of Internal Medicine, University of Utah School of MedicineSalt Lake City, Utah, 84132

**Keywords:** Alpha7, auditory system, cochlear, development, mouse, nicotinic acetylcholine receptor

## Abstract

Nicotinic acetylcholine receptor alpha7 expression was examined in the developing and adult auditory system using mice that were modified through homologous recombination to coexpress either GFP (alpha7GFP) or Cre (alpha7Cre), respectively. The expression of alpha7GFP is first detected at embryonic (E) day E13.5 in cells of the spiral prominence. By E14.5, sensory regions including the putative outer hair cells and Deiters' cells express alpha7GFP as do solitary efferent fibers. This pattern diminishes after E16.5 in a basal to apex progression, as Hensen's cells and cells of the spiral ligament acquire alpha7GFP expression. At birth and thereafter alpha7GFP also identifies a subset of spiral ganglion cells whose processes terminate on inner hair cells. Efferent fibers identified by peripherin or calcitonin gene-related protein do not coexpress alpha7GFP. In addition to cochlear structures, there is strong expression of alpha7GFP by cells of the central auditory pathways including the ventral posterior cochlear nucleus, lateral lemniscus, central inferior colliculus, and the medial geniculate nucleus. Our findings suggest that alpha7 expression by both neuronal and non-neuronal cells has the potential to impact multiple auditory functions through mechanisms that are not traditionally attributed to this receptor.

## Introduction

Numerous neurotransmitter systems contribute to the normal development and function of the auditory sensory (cochlear) apparatus and the circuitry of the central nervous system. This includes members of the excitatory ligand-activated nicotinic acetylcholine receptor family (nAChR; Albuquerque et al. 2009). The nAChR subunit family consists of 16 distinct subunits that in various pentameric combinations form ligand-activated ion channels that each exhibit uniquely specialized pharmacological and functional properties (Albuquerque et al. 2009). One of these is the homomeric alpha7 nAChR (α7) whose functional uniqueness is in part due to its expression by both neuronal and non-neuronal cells in many tissues throughout the body and because it is responsive to multiple agonists (including acetylcholine and choline as well as nicotine). This results in its ability to modulate a diverse range of cellular functions including cell growth, cell survival, neurotransmission, and inflammation (Gahring and Rogers 2005; Levin et al. 2006; Albuquerque et al. 2009).

Members of the nAChR family contribute to essentially all aspects of the auditory sensory system function and development (Morley and Happe 2000; Morley 2005). This includes widespread changes in expression during embryogenesis that optimizes their contribution to signal transduction, fine-tuning of sensory hair cells, and modulating central auditory circuit neurotransmission (Elgoyhen et al. 1994, 2001a; Happe and Morley 1998; Vetter et al. 1999, 2007; Morley and Happe 2000; Katz et al. 2004; Morley 2005). This functional diversity is in part accomplished through strict spatiotemporal control of different nAChR subunit expression, as has been extensively described for the nAChRs composed of either homomeric (α9) or heteromeric (α9 + α10) subunits (Elgoyhen et al. 1994; Vetter et al. 1999, 2007; Elgoyhen et al., 2001b; Murthy et al. 2009). Less is known about the role of other nAChRs including α7, although this receptor is implicated in modifying longer lived stimulation by high-frequency sound and supporting survival of spiral ganglion cells during development (Morley and Happe 2000; Morley 2005). Because the measurement of α7 expression and function can be compromised by low receptor expression levels or the absence of conditions that best reveal its modulatory role (Gahring and Rogers 2005; Albuquerque et al. 2009), the participation by this receptor as an important contributor to the development and normal auditory sensory function remains to be fully explored.

In this study, we examine α7 expression during development of the auditory sensory system. This was done using mice that were modified though methods of homologous recombination (Rogers and Gahring 2012; Rogers et al. 2012) to introduce, at the α7 gene 3′ end, a hemagglutinin (HA) protein tag to the α7 receptor protein and a bicistronic IRES-driven tau + enhanced-GFP fusion protein reporter (α7^GFP^). An advantage of the tauGFP reporter construct is that the tau component directs GFP into the axon of cells expressing α7^GFP^. Also generated was a mouse in which Cre-recombinase replaces the tauGFP. The expression of α7^GFP^ in these mice reveals extensive spatial and temporal remodeling of receptor expression during embryonic and postnatal development of the cochlear sensory structures. Furthermore, α7^GFP^ expression continues in both neuronal and non-neuronal cells of the adult cochlear structure and the central ascending auditory pathway. This suggests that α7 has the potential to impact functionally on auditory processes through multiple pathways and mechanisms that could impact upon the adult function in ways not traditionally attributed to this receptor.

## Materials and Methods

### Animals

All animals were used and housed in accordance with protocols approved in advance by the Institutional Animal Care and Use Committee at the University of Utah (Protocol Number (09-07003). This includes adherence to the Guide for the Care and use of Laboratory Animals of the National Institutes of Health.

### Generation of alpha7-HA-IRES-tauGFP and alpha7-HA-IRES-Cre mice

**Figure 1:**
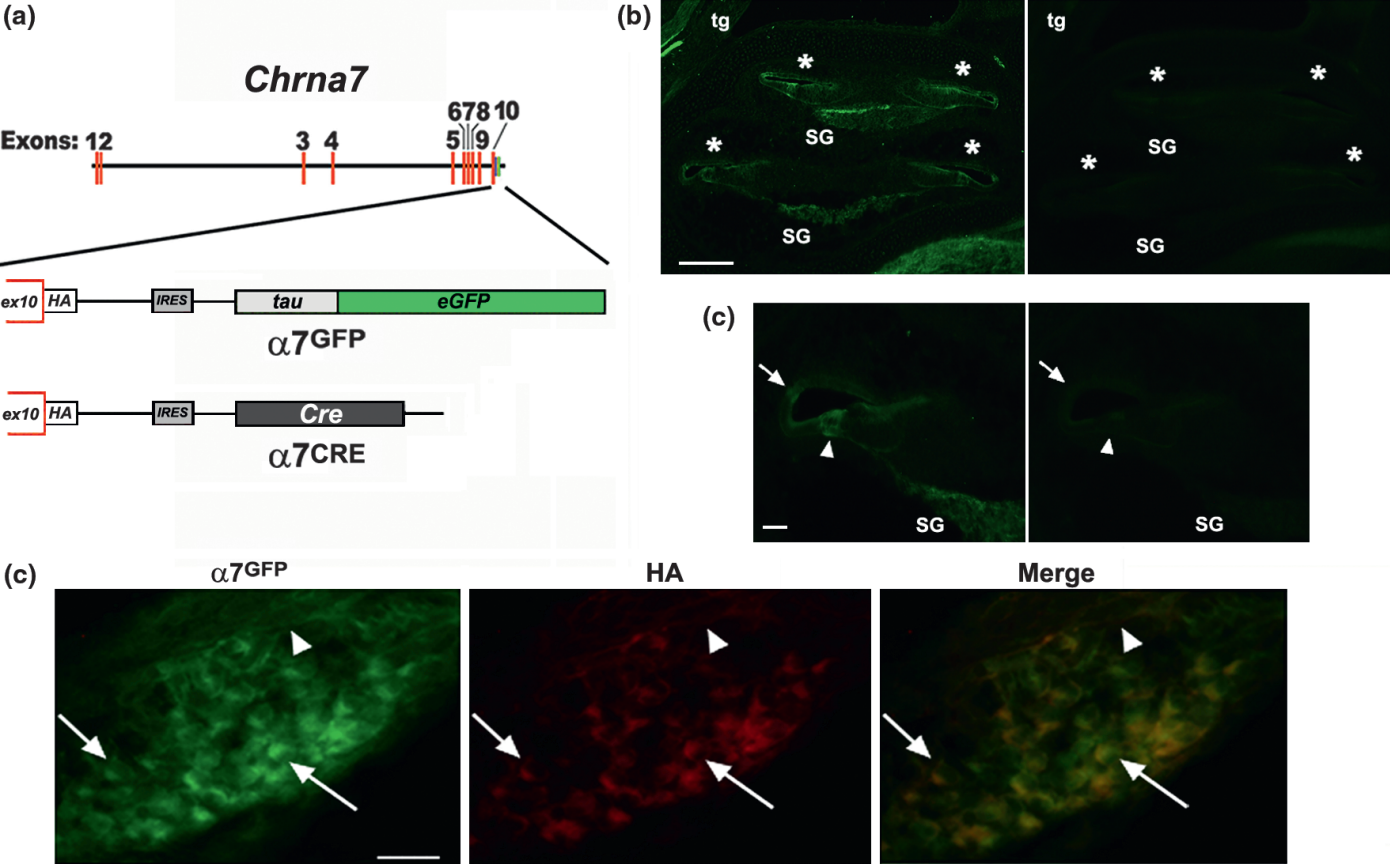
Mouse models used to examine nicotinic receptor α7 expression. (A) A diagram showing how the α7 gene (*Chrna7*) was modified using homologous recombination to add a C-terminus epitope tag (hemagglutinin [HA]) and inserted into the 3′ terminus of *Chrna7* a reporter bicistronic internal ribosome entry sequence (IRES)-tau fusion to enhanced green fluorescent protein (eGFP) fusion protein cassette (α7^GFP^; see Methods and Rogers and Gahring 2012; Rogers et al. 2012). This construct was subsequently altered by replacing the tau:GFP cassette with the Cre-recombinase gene (α7^Cre^). (B, C) The visualization of the *Chrna7* transcription using immunological detection of GFP compared with background. Shown are sagittal sections of the cochlear sensory structures of an E16.5 α7^GFP^ embryo in (B) and at greater magnification in (C). The panels on the left are stained for GFP expression (see Methods), whereas the image on the right shows an adjacent serial section that received the same staining treatment, only primary antibody was omitted. Photographs were collected at the same gain and exposure. The asterisk identifies cochlear ducts and the arrow points to the spiral prominence and the arrow head points to cell giving rise to the outer hair cells and Deiters' cells. Abbreviations are SG, spiral ganglion; and tg, trigeminal ganglion. In (B), the bar = 100 μm and in (C), the bar = 400 μm. (D) Examples of colabeling for α7^GFP^ (green) and anti-HA (HA) in cells associated with the spiral ganglion at E16.5. Examples of double-labeled cells are identified by with arrows. Some processes are also colabeled (arrow head). Bar = 50 μm.

The construction of the α7 protein and gene (*Chrna7)* reporter mouse lines; *Chrna7*-HA-IRES-tauGFP (α7^GFP^) and C*hrna7-HA-IRES-Cre* (α7^Cre^) have been described in detail (Rogers and Gahring 2012; Rogers et al. 2012). Briefly, as diagramed in Fig. 1A, the methods of homologous recombination were used to introduce an epitope hemagglutinin (HA) and stop codon extension to the α7 C-terminus and a bicistronic IRES-tauGFP reporter cassette (Rogers and Gahring 2012; Rogers et al. 2012). This generated the α7^GFP^ mouse (Fig. 1A), which expresses the tauGFP protein as a marker of *Chrna7* transcription. The Speed Congenic Program of the Jackson Laboratory was used to achieve 98% C57BL/6 background congenicity (Rogers et al. 2012). For conditional cell ablation of the cells expressing Cre as in the α7^Cre^ mouse, we crossed this mouse with the LoxP conditional diphtheria toxin (DTA) mouse lines as described previously (Rogers et al. 2012).

### Immunohistochemistry and microscopy

Embryo (E) timing was based upon identification of coital plugs (equal to E0.5). Immunohistochemical methods were as described (Rogers and Gahring 2012; Rogers et al. 2012). Embryos were fixed in PBS/2% paraformaldehyde/5% sucrose, cryoprotected with sucrose in PBS to a final of 30%, embedded and sectioned using a Microm EM550 microtome. The 12-μm sections were mounted on glass slides, blocked, and permeabilized with 1% deoxycholate and 0.2% Triton X-100 in PBS, and then incubated overnight at 4°C with the appropriate primary antibodies. After washing, sections were incubated with secondary antibodies conjugated to fluorescent markers (Jackson ImmunoResearch, West Grove, Pennsylvania) for 1 h at room temperature. The sections were again washed, and mounted in prolog gold antifade reagent (Invitrogen, Grand Island, New York; P36930) and cover-slipped before being photographed using fluorescence microscopy (Rogers et al. 2012). Images were collected using a Microfire 24-bit CCD camera (Optronics, Goleta, California) and imported into Photoshop C2 for preparation of figures.

The antibodies used were commercially obtained. These were anti-calcitonin gene-related protein (CGRP; rabbit; 1:30; Chemicon/Millipore, Temecula, Californa AB5920), anti-GFP (chicken; 1:800, Aves Labs, Tigard, Oregon GFP-1020), anti-HA (rabbit; 1:200; HA.11 Covance, Princeton, New Jersey PRB-101P), anti-peripherin (rabbit; 1:100; Abcam, Cambridge, Massachusetts #1530), anti-S100beta (rabbit; 1:100; Abcam ab868), rabbit anti-beta-III tubulin (TUJ1; 1:3000; Covance MMS-435P). Detection of GFP offers superior sensitivity that is well over background fluorescence (Fig. 1B and C). For this study, some inconsistent signal detection or autoflourescence was occasionally observed and these sites identified in the individual figures. We find the expression of GFP and HA are similar, although anti-HA expression is detected predominantly on the surface of cells identified by anti-GFP expression (Fig. 1D).

## Results

**Figure 2:**
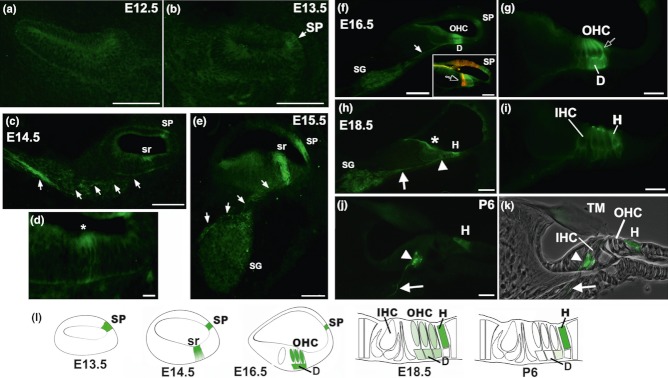
The expression of α7^GFP^ varies with cochlear development. The expression of α7^GFP^ identified by immunohistochemical detection of GFP (Methods) is shown for the embryonic cochlear structures in the sagittal section plane. (A) E12.5 shows one early cochlear structure and associated background fluorescence. (B) E13.5, the first identified expression of α7^GFP^ in cells of the spiral prominence (sp). (C) An E14.5 cochlear structure shows the expression of α7^GFP^ in the presumptive sensory region (sr) and pioneering efferents are also identified (arrows). (D) The E14.5 sr is shown at greater magnification. (E) E15.5 increased expression of α7^GFP^ by cells of the sr is present as ar sp cells. Labeled processes associated with the spiral ganglion (SG) are identified by (arrows). (F) E16.5 α7^GFP^ is seen in outer hair cells (OHC) and Deiters' cells (D). The expression of α7^GFP^ in afferent processes is noted (arrow). The insert box shows a similar section colabeled with α7^GFP^ (green) and S100beta (red; Buckiova and Syka 2009) identifies the inner hair cell (open arrow). (G) Increased magnification of the E16.5 sensory region showing the OHC and Deiters' (D) cell labeling (open arrow). (H) The E18.5 cochlear structure exhibits decreased α7^GFP^ expression in OHCs and acquisition of label by Hensen's cells (H). Also labeled are SG afferents (arrow) and their terminals on the base of IHCs (arrow head). The asterisk identifies autofluorescence in the tectorial membrane that is inconsistently present. (I) The E18.5 sensory region is shown at greater magnification. (J) The postnatal day 6 (P6) α7^GFP^ expression pattern reveals the SG afferents (arrow) that terminate as part of the extensive synaptic terminals at the base of the IHCs (arrow head). A Hensen's cell (H) is identified. (K) The P6 α7^GFP^ expression image in Panel I is superimposed over a phase contrast image of the cochlear structure. Single afferents (arrow) and their terminals (arrow head) are evident. Tectorial membrane (TM). (L) Diagrams depicting the adult cochlear structures (as labeled) and the major expression patterns of α7^GFP^ (green) at the corresponding developmental stage. Bars = 100 μm (A, B, C, E, F, H) or 20 μm (D, F-insert,G, I).

The expression of α7 exhibits distinct spatiotemporal patterning in developing cochlear structures. Previously, we demonstrated the earliest expression of α7 in the developing embryo to be in rhombomeres 3 and 5 of the E9.0 embryo (Rogers et al. 2012). Thus, we initiated studies of α7^GFP^ staining at this time. From E9.5 through approximately E12.5, the otic and cochlear structures did not express detectable α7^GFP^ (Fig. 2A and not shown, see Rogers et al. 2012). The earliest detected expression of α7^GFP^ in the cochlear structures was at E13.5 in cells of the spiral prominence (SP; Fig. 2B). The SP retains α7^GFP^ expression throughout embryonic and post-natal development (see below). By E14.5 (Fig. 2C and D), α7^GFP^ expression extends to cells in the sensory domain of the lesser epithelial ridge near the site of the presumptive outer hair cells (OHC) and Deiters' cells (Morsli et al. 1998; Lanford et al. 1999; Kiernan et al. 2005a,b). Light staining of the greater epithelium ridge was also present from E14.5 and thereafter, although this staining is inconsistently observed (Fig. 1B and C and not shown). Coincident with this expression was strong staining of pioneering efferents that become separated into individually distinguished processes as they progress through the spiral ganglion (SG) to reach the external face of this sensory domain (Fig. 2C; see below). The staining of the epithelial cells of the lesser epithelial ridge intensifies thereafter (e.g., E15.5 in Fig. 2E). At this stage, expression of α7^GFP^ by cells of the SG was in general only weakly observed in scattered cells (Fig. 2E). By E16.5, α7^GFP^ expression continues to increase in cells of the lesser epithelial ridge of the prosensory domain where OHC and Deiters' cells can now be distinguished (Fig. 2F and G and insert). Cells throughout the SG were also revealed by expression of α7^GFP^ by this developmental stage. Pillar cells do not express α7^GFP^ and there were no identifiable efferent processes labeled by the expression of this receptor at this stage or thereafter (see the following sections).

The pattern of α7^GFP^ expression in the E18.5 cochlear structure undergoes significant remodeling as both sensory hair cells and the associated supporting cells complete their differentiation (Fig. 2H and I). This includes a decrease of α7^GFP^ expression by OHCs and underlying Deiters' cells that progresses away from the inner hair cells and proceeds in a basal-to-apical direction (next section). This is coincident with the appearance of signal in Hensen's cells that are most proximal to the outer line of OHCs (returned to below). Ganglionic afferent fibers ending at the base of the inner hair cells are also detected (see subsequent sections). In the postnatal mouse, as shown in the P6 cochlear sensory structure (Fig. 2J and K), the expression of α7^GFP^ becomes limited to Hensen's cells immediately adjacent to the most distal OHC. Cells of the spiral ligament also acquire α7^GFP^ expression, while the spiral prominence remains unchanged. In the SG, the expression of α7^GFP^ is well established and the projections from these labeled cells can be followed to the vicinity of the inner hair cells (IHC) where their terminals appear to surround the base of the inner hair cell (IHC; Fig. 2J and K). A summary diagram illustrating the expression of the α7^GFP^ during these major developmental stages is shown in Fig. 2L.

### Remodeling of α7^GFP^ in the cochlear structure after E16.5 is in a basal-to-apical direction

**Figure 3:**
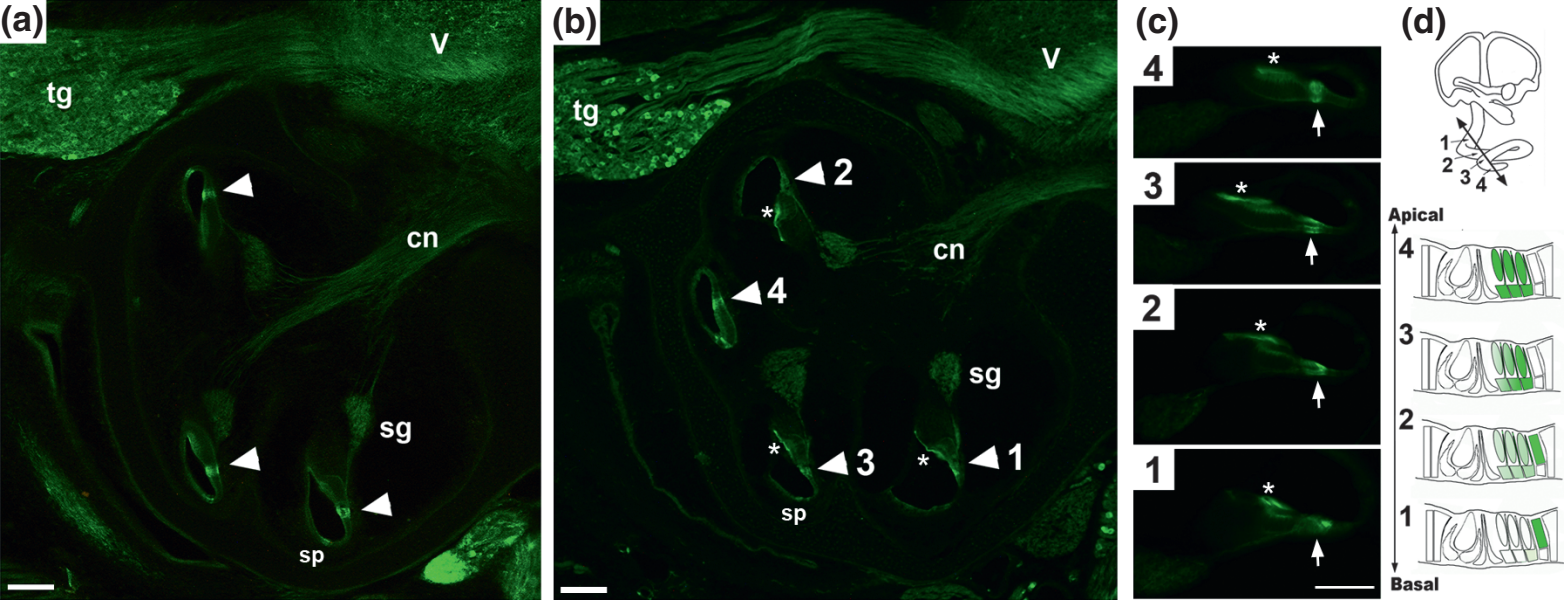
Remodeling of α7^GFP^ Expression is from basal to apical. (A) A sagittal section showing the E16.5 cochlear structure and α7^GFP^ expression (green). At this stage, all cochlear structures exhibit a similar pattern of α7^GFP^ expression by cells of the lesser epithelial ridge and presumptive sensory region (arrow heads). Also labeled are cells of the SG, the cochlear nerve (cn), trigeminal ganglion (tg), and the trigeminal nucleus (V) and spiral prominence (sp). (B) A similar view of the E18.5 cochlear structure shows four cochlear ducts (arrow heads) that are numbered in a basal-to-apex direction. (C) Greater magnification of the numbered cochlear ducts in B shows the corresponding α7^GFP^ expression. The arrow indicates the site of shift in α7^GFP^ expression. The asterisks identify autofluorescence in the tectorial membrane. (D) Diagrams illustrating the approximate site of each numbered duct in the cochlear structure and identification of the α7^GFP^ labeling of the individual cochlear sensory cells regions. Bars = 100 μm.

The remodeling of the sensory cell region of the cochlear structure between E16.5 and E18.5 as suggested by the progression in changing α7^GFP^ expression was examined further. Through E16.5, all otic structures exhibit a similar α7^GFP^ expression pattern (Fig. 3A). This was not the case in the E18.5 cochlear structure where the loss of α7^GFP^ expression by OHC and Deiters' cells and acquisition of staining by Hensen's cells was first observed in the most basal structures and it then appears in the more apical structures successive developmental stages (Fig. 3B and C and not shown). This generates a striking contrast in α7^GFP^ expression between cochlear structures at the apex relative to the base with intermediary turns, exhibiting the progressive stages of this change in α7^GFP^ expression (Fig. 3B). By P4, this gradient was not evident (not shown) and the mature α7^GFP^ expression pattern first observed in the E18.5 basal cochlear structures was present across the entire structure. In Fig. 3D, a diagram depicts the remodeling of α7^GFP^ expression seen in the E18.5 developing cochlear structure.

### Nonsensory cells of the cochlear structure express α7^GFP^

**Figure 4:**
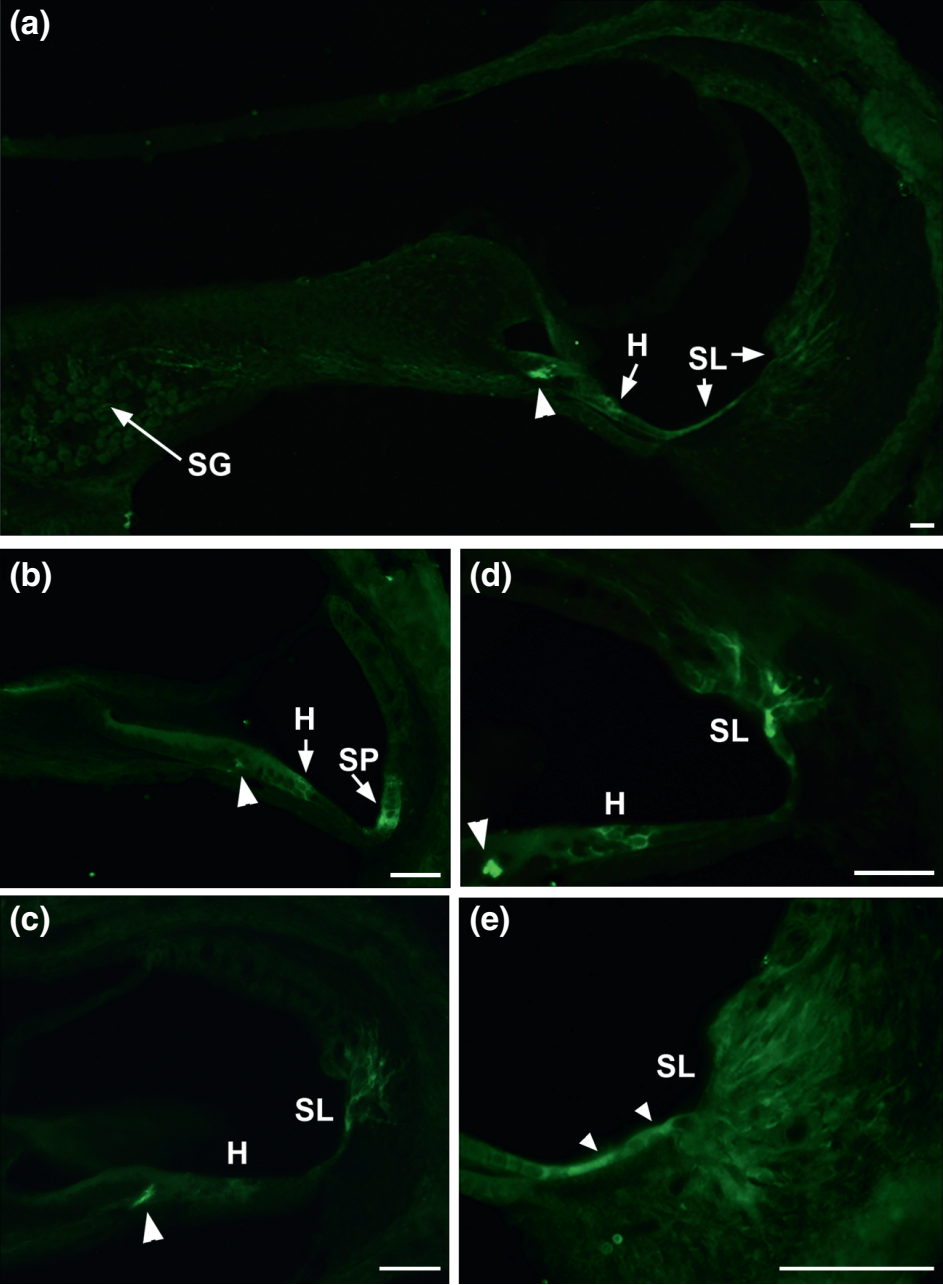
Postnatal expression of α7^GFP^ in the cochlear structure. (A) An image of a sagittal section showing the P6 cochlear structure and the expression of α7^GFP^ in afferents originating from spiral ganglion (SG) cells that terminate (arrow head) at the base of the inner cell. Hensen's cells (H) and cells in the spiral ligament are also labeled (SL). (B) A low-magnification image of an adjacent section shows the expression by cells of the spiral prominence (SP). Other labels are as in (A). (C) Another serial section reveals expression of α7^GFP^ in cells of the spiral ligament (SL). (D) Increased magnification shows the α7^GFP^ signal to be in SL cell bodies and the elaborated processes of these cells. (E) SL cell bodies in the P12 mouse. The labeled cell bodies (arrow heads) and their associated processes extend throughout this region.

As suggested by the preceding discussion, there was expression of α7^GFP^ by both neuronal and non-neuronal cells (Fig. 4). This is particularly clear in the postnatal mouse (e.g., P6–P12), where the predominant expression of α7^GFP^ in neuronal cells was by cells of the SG (Fig. 4A). The strongest labeling of cochlear structures was restricted to Hensen's cells and the spiral prominence (Fig. 4A–E). Evident at the P6 stage was α7^GFP^ signal in individual cells of the spiral ligament (Fig. 4C and D). Also evident were the extended branching that is characteristic of the morphology of type II fibrocytes located in this region (Fig. 4D; Spicer and Schulte 1991; Sun et al. 2012). In the P12 cochlear structure, the branches were more abundant and form a ‘feathered’ structure that emanates from cell bodies defined by α7^GFP^ expression (Fig. 4E). Cells of the stria vascularis or other members of the cell family composing the structures of the lateral wall and surrounding cochlear duct were not observed to express α7^GFP^ in these later stages of development (Fig. 4).

### The expression of α7^GFP^ during innervation of the developing cochlear structure

**Figure 5:**
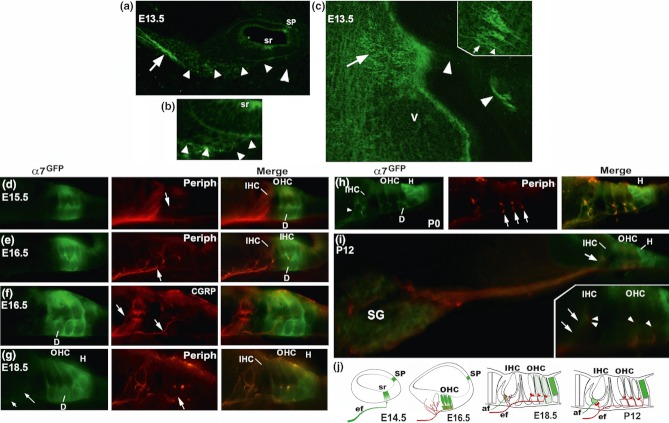
The α7^GFP^ expression during cochlear innervation. Innervation of the developing cochlear structure is revealed by α7^GFP^ labeling. (A) An E13.5 sagittal section shows a group of efferent processes (arrow) that distribute to solitary fibers that are strongly labeled for α7^GFP^ expression (arrow heads). Cells of the putative sensory region (sr) and the spiral prominence (SP) are identified. (B) At greater magnification, these fibers (arrow heads) have a beaded appearance and project towards the base of sr. (C) The possible origin of the pioneering efferent fibers is suggested by the intense expression of α7^GFP^ in the E13.5 cell groups (arrow) located caudal to the trigeminal sensory nucleus (V) consistent with the early olive in this horizontal section through the posterior brain stem. At increased magnification (Insert), the cell clustering (arrow) and their projections (arrowhead) are identified. Serial sections (not shown) reveal continuity between these cells and those entering the cochlear structures (arrow heads). (D) E15.5 α7^GFP^ expression and colabeling with other neuronal process markers (red). The processes that express peripherin (arrow) end mostly in the vicinity of the inner hair cells (IHC). Occasional solitary fibers (arrow) extend towards the base of the outer hair cells (OHC) at the dorsal boarder of the Deiters' cells (D). (E) The E16.5 cochlear innervation pattern looks much the same as E15.5, although the peripherin-labeled fibers (arrow) are more distinct. These processes lack detectable α7^GFP^ expression. (F) Olivocochlear efferents identified by calcitonin gene-related proteins (CGRP; arrows). (G) The E18.5 embryo exhibits afferents detected by α7^GFP^ expression (arrows). These extend from SG cells that are not colabeled with peripherin (not shown). (H) At birth (P0), there are distinctly labeled α7^GFP^ afferents (arrowhead) and peripherin-labeled efferents that extend to the Deiters' cells (D) and then turn (arrows) to contact the base of the OHCs. Hensen's cells are noted (H). (I) The P12 innervation pattern is similar to the P0. In this merged image of α7^GFP^ expression (green) and peripherin (red), many spiral ganglion (SG) cells and processes are labeled, but the labels only rarely overlap in the same processes (see insert). The α7^GFP^ identify mostly processes reaching the IHCs (arrow). Peripherin-labeled processes mostly terminate at the base of the outer hair cells (OHC) or onto the α7^GFP^-labeled afferent fiber near the base of the IHC. Hensen's cells expressing α7^GFP^ is identified (H). The inset shows the sensory cell region at increased magnification. The arrows identify the α7^GFP^-expressing afferent ending at the base of nonlabeled IHC, whereas the double arrow heads point to the peripherin-labeled terminal. Other peripherin processes extend to the base of the OHCs (individual arrow heads). (J) Diagrams as in Fig. 2 depicting the basic innervation patterns observed in this study. Green is α7^GFP^ and red is peripherin. Afferent (af) and efferents (ef). Bars = 50 μm

Innervation of cochlear sensory cells follows a series of distinct steps that were in part revealed by α7^GFP^ visualization (Fig. 5). As noted, the first detection of α7 expression was in the prominently labeled efferent processes that appear to form bundles upon entering the SG and then disperse into small solitary fibers (E14.5; Fig. 5.A and 2C,D). These solitary processes exhibit a beaded structure as they proceed to the base of the developing sensory cells (Fig. 5B).

The origin of these efferent fibers was examined in serial sections of the E14.5 hind brain. These fibers appear to originate from a cell grouping in the basal brain stem caudal to trigeminal nucleus V that could be distinguished by their transient α7^GFP^ expression (Fig. 5C). These cells occur in clusters (Fig. 5C insert) and their prominently labeled processes can be followed using serial section sets to the cochlear structure where they give rise to the fiber bundles and the point of dissemination associated with the SG (Fig. 5C and insert). The anatomical location of these cells suggest that these cells are within the forming olive complex, which is consistent with the reports of pioneering fibers that originate from the developing olive complex and extend to the developing cochlea (Zuo et al. 1999). These fibers were not detected after E15.5.

During the E15.5–16.5 period, there was essentially no labeling of neuronal processes by α7^GFP^ (Fig. 5D–F). However, ongoing innervation of cochlear sensory cells was identified using peripherin labeling (Fig. 5E; see Simmons et al. 1996; Hafidi 1998; Huang et al. 2007) or for olivocochlear efferents that were identified by labeling for calcitonin gene-related protein (CGRP; Fig. 5F, Fritzsch 2003). By E18.5, the SG α7^GFP^ signal was present in afferent processes that extend to the base or near vicinity of the IHCs (Fig. 5G).

At birth and thereafter (P0–P12 analyzed), the expression of α7^GFP^ was strongly detected in SG afferent fibers where they terminate near or at the base of IHC sensory cells (Fig. 5H and I). This basic pattern of α7^GFP^ expression was reinforced during the remaining postnatal period as fibers continue to form a dense plexus that appears to surround the base of the IHCs. The other efferent fibers not detected by α7^GFP^ continue to be trimmed and also associate with their final targets (Merchan-Perez and Liberman 1996; Simmons et al. 1996; Hafidi 1998; Huang et al. 2007). The outcome of this remodeling was evident by P12 when the SG1 afferent terminals surrounding the IHC were distinguished by strong α7^GFP^ staining of the terminal clusters (Fig. 5I and inset). This was approximately the same time hearing onset occurs in mice (∼P10; Kros et al. 1998). Processes originating from SG cells identified by peripherin expression that were not colabeled with α7^GFP^ form distinct efferent terminals on or very near OHCs cells and on the terminals that end on the IHC afferent terminals identified by α7^GFP^ labeling (Fig. 5I; Huang et al. 2007). While not entirely evident from the images shown, not all SG cells at P12 expressed α7^GFP^, suggesting this could identify a functionally distinct subpopulation (Fig. 5I; Happe and Morley 1998). Again, no α7^GFP^ labeling of olivocochlear efferents was detected. A diagram summarizing these findings is shown in Fig. 5J.

### Ablation of the α7^Cre^-expressing cell lineage confirms α7^GFP^ expression during cochlear development

**Figure 6:**
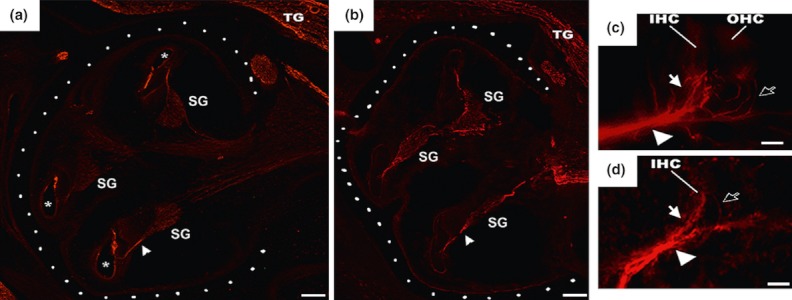
The ablation of α7 cell lineages is consistent with α7^GFP^ expression. Comparison of a cochlear structure labeled for expression of the filament marker peripherin from an E16.5 α7^GFP^ mouse (A) and similarly timed α7^Cre:^^DTA^ ablated embryo (B). The basic patterning of the cochlear structures is outlined by white dots and the spiral ganglia (SG) and trigeminal nerve (TG) are identified, Arrow heads point to cochlear nerves and the asterisk identifies in A the cochlear ducts (asterisk in A). (C) Greater magnification of the cochlear sensory cell region of an α7^GFP^ or (D) α7^Cre:^^DTA^ embryo. The arrow heads identify prominent cochlear nerve bundles and filled arrows point to fibers in the inner cell (IHC) region. Open arrows identify fibers extending to the sensory cell region, giving rise to outer hair cells and Deiters' cells. Bars: 100 μm (A, B); 20 μm (C, D).

Although α7^GFP^ expression was not detected in the developing cochlear structures until E13.5 (Fig. 2B), as reported previously the earliest α7 expression we have defined is at P9.0 in rhombomeres 3 and 5 (Rogers et al. 2012). Because cochlear morphogenesis includes signaling from rhombomere 5 (Liang et al. 2010), the possibility of α7^GFP^ contributing to the development of this structure was examined. This was done using embryos from α7^Cre^ mice crossed with mice harboring the conditional ROSA26-loxp (diphtheria-A toxin (DTA; termed α7^Cre:DTA^; Rogers and Gahring 2012). In these embryos, α7^Cre:DTA^-expressing cells and their direct lineages were ablated, thus revealing expression that could have been be missed by α7^GFP^ measurements (Rogers and Gahring 2012). An example of the cochlear structure at E16.5 taken from α7^Cre:DTA^ crosses is shown in Fig. 6. Because there is only occasional overlap with α7^GFP^ (see Fig. 5E), we used peripherin expression to aid in examining the fate of non-α7-expressing cells (Fig. 6A and B). The overall patterning of the cochlear structure and the formation of major boney structures of the cochlea inclusive of the otic capsule and modiolus were intact, albeit somewhat distorted. The cochlear ducts were collapsed (Fig. 6B), probably due to the absence or severe thinning of the distal lateral wall. Also absent was the sensory cell domain containing presumptive OHCs and Deiters' cells (Fig. 6C and D), as expected from results of α7^GFP^ expression (Fig. 2., 5).

The SG of α7^Cre:DTA^ embryos is reduced in size and the majority of cells remaining give rise to mostly peripherin-labeled efferents (see Fig. 5E). These fibers also appear to be more densely aggregated relative to the α7^GFP^ control mouse (Fig. 6A and B). While peripherin-identified processes still project to the presumptive sensory cells (both IHC and OHC), they were less branched and those that did project to the former OHC target fields often turn and proceed backwards towards the vicinity of IHCs (Fig. 6C and D). These results are consistent with the earliest expression of α7 being after major cochlear structures are determined, and there was the expected selective ablation of OHCs and Deiters's cells. The necessity of the presence of the target sensory cell to coordinate the innervation process is also suggested by these findings.

### Auditory pathways in the postnatal central nervous system are identified by α7^GFP^ expression

**Figure 7:**
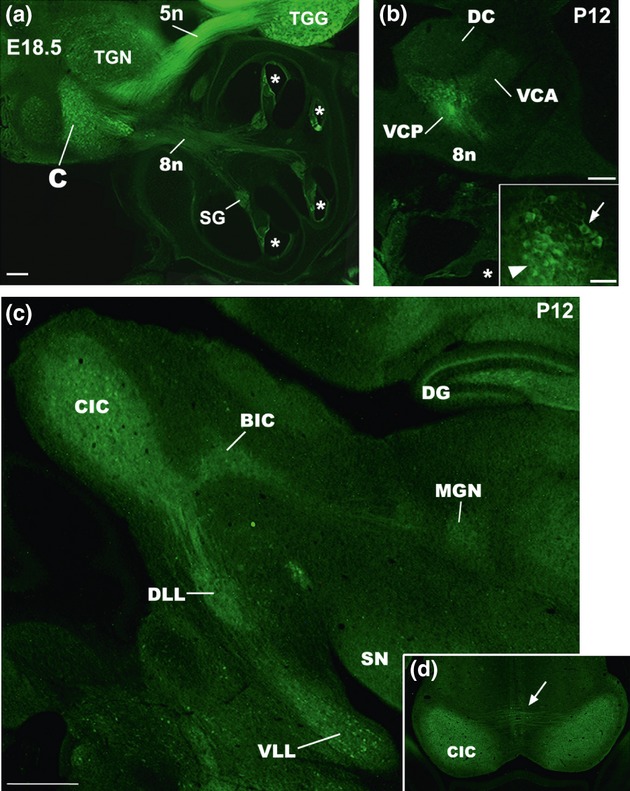
Central auditory systems express α7^GFP^. Central auditory nuclei identified by α7^GFP^ expression. (A) At E18.5 in this sagittal image of the entire otic complex and the adjacent basal brainstem is included. The cochlear nucleus (C) and the eighth cranial nerve (8n) are visible as is the fifth cranial nerve (5n), the trigeminal nucleus (TGN), and trigeminal ganglion (TGG). Also noted are cochlear ducts (asterisk) and a spiral ganglion (SG). (B) At P12, α7^GFP^ expression of cochlear complex reveals the strongly labeled cells of the ventral-posterior cochlear nucleus (VCP). The dorsal cochlear nucleus (DC) and ventral-anterior cochlear nucleus (VCA) are identified and is the eighth nerve (8n) and a cochlear duct (asterisk). The inset shows the VCP at increased magnification. Cells clusters expressing α7^GFP^ (arrowhead) and individual cells that resemble the morphology of octopus cells described previously (Morley and Happe 2000; Morley 2005) to express a7 (arrow) are noted. (C) Another P12 sagittal section reveals α7^GFP^ expression in the ascending central auditory pathways. (D) The expression of α7^GFP^ in the inferior colliculus (CIC) of this horizontal section reveals staining of the commissural fibers (arrow). Structures identified are the brachium of the inferior colliculus (BIC); dentate gyrus (DG), inferior colliculus, central nucleus (CIC); lateral lemniscus, dorsal nucleus (DLL); lateral lemniscus, ventral nucleus (VLL); medial geniculate nucleus (MGN), and the substantia nigra (SN). Bars = 100 μm (A, B0; 20 μm (B-insert), and 1 mm (C, D).

The results of studies examining α7 expression using in situ hybridization and functional measurements using electrophysiology have shown that this receptor is an important contributor to various nuclei of the central auditory system (Happe and Morley 1998; Vetter et al. 1999, 2007; Morley and Happe 2000; Morley 2005). The α7^GFP^ mouse system offers an excellent opportunity to view these central systems and their connections as shown in Fig. 7. The connections between the SG and the cochlear nuclei were strongly identified at E18.5, presumably due to the dense projections from SG cells expressing α7^GFP^ that extend processes both to the IHC (Fig. 2) and the developing cochlear nuclei of the brainstem (Fig. 7A).

The expression of α7^GFP^ appears to intensify after P10, and by P12 signal is consolidated almost exclusively in the ventral-posterior cochlear nucleus (Fig. 7B). This is in agreement with reports from in situ hybridization studies reporting the strong expression of α7 in this nucleus, whereas other major cochlear nuclear divisions exhibited only weak or sporadic labeling (Yao and Godfrey 1999; Morley and Happe 2000). Also consistent with those studies was that the cells identified by α7^GFP^ expression resemble octopus cells (Fig. 7B, insert). Essentially, no expression of α7^GFP^ was detected in the dorsal cochlear nucleus, although some dispersed and weakly stained cells were present in the granular aspect. Also evident was the strong staining of neuropil, presumably in part due to terminals of SG cells associated with the eighth cranial nerve (Fig. 7B, inset). This strong labeling of the P12 SG and OHC afferents is consistent with other reports (Morley and Happe 2000).

The expression of α7^GFP^ also persists into the adult animal. This is apparent in the ascending central auditory system nuclei and their fibers (Fig. 7C). After the cochlear nucleus, α7^GFP^ is present in the ventral lateral lemniscus, on through the dorsal lateral lemniscus, and to the inferior colliculus where dense staining of α7^GFP^ is present (Fig. 7C; Morley and Happe 2000; Yao and Godfrey 1999). The commissural fibers of the inferior colliculus are also identified by α7^GFP^ expression (Fig. 7D). Thereafter, efferents follow the brachium of the inferior colliculus to the medial geniculate nucleus where scattered cells expressing α7^GFP^ were seen. Not shown is that the expression of α7^GFP^ in the adult auditory cortex appears restricted to cells of layer 1. Labeling of olivocochlear fibers was not detected.

## Discussion

This study extends the reports of spatiotemporal regulation of α7 expression during mouse embryonic development (Rogers and Gahring 2012; Rogers et al. 2012) to include the cochlear sensory structure, as well as confirms the extensive expression of this nAChR in the ascending central auditory system. The novel finding that in addition to expression of α7^GFP^ in developing sensory cells of the cochlear structure and neuronal cells of the spiral ganglion, there is also considerable expression by nonsensory cells. Cells of the spiral prominence and ligament, Deiters' cells, and some Hensen's cells. Despite overall agreement between our studies and those using in situ hybridization (e.g., Happe and Morley 1998; Morley and Happe 2000), these nonsensory cells were not reported previously to express α7. However, these comparisons are incomplete because the earlier studies did not necessarily show the comparable structures or the developmental stages at times where we observed peak α7^GFP^ expression. Also, our method of detecting GFP as a marker of α7 expression offers improved sensitivity and resolution that has previously not been available for this nAChR.

The nicotinic receptors α9 and α10 are particularly well characterized in the auditory system (Elgoyhen et al. 1994, 2001a; Vetter et al. 1999, 2007; Katz et al. 2004; Morley 2005). Comparing the expression of α7^GFP^ to the results from these studies of the sensory hair cells and the nonsensory cells of the cochlea indicate that there are significant spatiotemporal differences during development between the expression of α7 versus α9 and/or α10. The α9^KO^ mouse also exhibits auditory deficiencies that are not observed in the α7^KO^ mouse, which is largely devoid of a phenotype in this sensory system under normal physiological conditions (Liberman and Brown 1986; Simmons and Morley 1998; Morley 2005; Lustig 2006). The α7^GFP^ is not detected in IHCs, which is consistent with α9 nAChR being the principle target of alpha-bungarotoxin in this cell type (Uziel et al. 1981; Glowatzki and Fuchs 2000). Collectively, this suggests that functional redundancy between these receptor subtypes is unlikely (see also Rogers and Gahring 2012). This is also supported by the extensive studies by the Morley group (Happe and Morley 1998, 2004; Morley and Happe 2000; Simmons and Morley 2011) who showed that multiple receptor subtypes are expressed in the cochlear and central auditory systems, but each exhibits distinct spatiotemporal patterns that likely preclude substantial or sustained functional overlap.

Noteworthy is that the functional contribution of α7 towards modulating physiological systems may not be revealed unless the system is imbalanced as by genetic deficiencies, sustained exposure to pharmacological compounds, or other events such as inflammation (e.g., Faustman et al. 1992; Gahring and Rogers 2005; Venables et al. 2007; Albuquerque et al. 2009; Brown 2011; Severance et al. 2011). For example, the dysfunction of α7 is implicated in several psychiatric syndromes associated with certain forms of autism and schizophrenia (particularly in patients who hallucinate) whose spectrum of disorders include abnormal sensitivity to sensory stimuli including an abnormal auditory gating phenotype (Khalfa et al. 2001; Veuillet et al. 2001; Araki et al. 2002; McEvoy and Allen 2002; Freedman et al. 2003; Lippiello 2006; Martin and Freedman 2007; Wallace and Porter 2011 and references therein). Also, the association of certain auditory deficits and nicotine abuse, mostly associated with cigarette smoking, has further focused speculation on the role of α7 in these pathologies and the possible advantages of therapeutically targeting this receptor for symptomatic relief in these cases (Araki et al. 2002; McEvoy and Allen 2002; Simosky et al. 2002; Freedman et al. 2003; Levin et al. 2006; Lippiello 2006; Martin and Freedman 2007; Wallace and Porter 2011). In this context, our results suggest additional lines of investigation. For example, in α^7Cre:DTA^ cell lineage ablation there are collapsed cochlear ducts and abnormal innervation indicating that the cells express α7 and the cells that do so contribute an obligatory role in the successful development and long-term function of these structures. The α7 receptor could also participate in auditory performance after birth, including functions related to the central auditory pathways. This study also adds the possibility of an effect by α7 on the performance of the spiral ligament. These cells exhibit a cholinergic response that is most often described in terms of muscarinic acetylcholine receptors (Khan et al. 2002; Maison et al. 2010), and their dysfunction is related to several pathogenic auditory deficiencies (Spicer and Schulte 1991; Slepecky et al. 1995; Kikuchi et al. 2000; Sun et al. 2012). The role of α7 has, to our knowledge, not been examined in these cells. Collectively, the potential for α7 functional pleiotropy in the auditory system is similar to other tissues we have recently examined (Rogers and Gahring 2012). Thus, multiple defects that impact upon adult function could be expected depending upon the timing, duration, and nature of the receptor dysfunction.
